# Incremental growth of therizinosaurian dental tissues: implications for dietary transitions in Theropoda

**DOI:** 10.7717/peerj.4129

**Published:** 2017-12-11

**Authors:** Khai Button, Hailu You, James I. Kirkland, Lindsay Zanno

**Affiliations:** 1 Department of Biological Sciences, North Carolina State University, Raleigh, NC, United States of America; 2 Paleontology, North Carolina Museum of Natural Sciences, Raleigh, NC, United States of America; 3 Key Laboratory of Vertebrate Evolution and Human Origins of Chinese Academy of Sciences, Institute of Vertebrate Paleontology and Paleoanthropology, Chinese Academy of Sciences, Beijing, China; 4 College of Earth Sciences, University of Chinese Academy of Sciences, Beijing, China; 5 Utah Geological Survey, Salt Lake City, UT, United States of America

**Keywords:** Dinosaur teeth, Therizinosauria, Von ebner, Dentin, Enamel microstructure, Histology, Tooth growth

## Abstract

Previous investigations document functional and phylogenetic signals in the histology of dinosaur teeth. In particular, incremental lines in dentin have been used to determine tooth growth and replacement rates in several dinosaurian clades. However, to date, few studies have investigated the dental microstructure of theropods in the omnivory/herbivory spectrum. Here we examine dental histology of Therizinosauria, a clade of large-bodied theropods bearing significant morphological evidence for herbivory, by examining the teeth of the early-diverging therizinosaurian *Falcarius utahensis*, and an isolated tooth referred to *Suzhousaurus megatherioides*, a highly specialized large-bodied representative. Despite attaining some of the largest body masses among maniraptoran theropod dinosaurs, therizinosaurian teeth are diminutive, measuring no more than 0.90 cm in crown height (CH) and 0.38 cm in crown base length (CBL). Comparisons with other theropods and non-theropodan herbivorous dinosaurs reveals that when controlling for estimated body mass, crown volume in therizinosaurians plots most closely with dinosaurs of similar dietary strategy as opposed to phylogenetic heritage. Analysis of incremental growth lines in dentin, observed in thin sections of therizinosaurian teeth, demonstrates that tooth growth rates fall within the range of other archosaurs, conforming to hypothesized physiological limitations on the production of dental tissues. Despite dietary differences between therizinosaurians and hypercarnivorous theropods, the types of enamel crystallites present and their spatial distribution—i.e., the schmelzmuster of both taxa—is limited to parallel enamel crystallites, the simplest form of enamel and the plesiomorphic condition for Theropoda. This finding supports previous hypotheses that dental microstructure is strongly influenced by phylogeny, yet equally supports suggestions of reduced reliance on oral processing in omnivorous/herbivorous theropods rather than the microstructural specializations to diet exhibited by non-theropodan herbivorous dinosaurs. Finally, although our sample is limited, we document a significant reduction in the rate of enamel apposition contrasted with increased relative enamel thickness between early and later diverging therizinosaurians that coincides with anatomical evidence for increased specializations to herbivory in the clade.

## Introduction

Previous research has identified functional and phylogenetic signals in the dental microstructure of many extant and extinct amniotes (Johnston, 1979). Of the four major tissue components of teeth (enamel, dentin, cementum, and pulp), dentin has received the most attention in recent years due to the presence and (in the case of fossil specimens) preservation of daily von Ebner lines (**VEL**s), which represent incremental tissue production and can be used to glean paleobiological data (Dean et al., 1993; Erickson, 1996a; Erickson, 1996b; D’Emic et al., 2013). For example, counting the number of VELs and measuring the average increment widths has yielded calculations of tooth growth and replacement rates in fossil hominids (Dean, 2006), dinosaurs (Erickson, 1996b; Sereno et al., 2007; D’Emic et al., 2013; García & Zurriaguz, 2016; Erickson et al., 2017), and other extinct taxa (Scheyer & Moser, 2011; Heckert & Miller-Camp, 2013). These trajectories can in turn be linked to a variety of macroevolutionary trends including gigantism in hypercarnivorous theropods (Erickson, 1996b) and the evolution of dental batteries in megaherbivorous ornithischians and sauropodomorphs (Upchurch & Barrett, 2000; Sereno et al., 2007; D’Emic et al., 2013; Barrett, 2014; Button et al., 2015; Erickson et al., 2015; García & Zurriaguz, 2016) to better understand the relationship between dentition and other facets of dinosaurian paleobiology. However, information on tooth growth can also be derived from cementum and enamel as these tissues also exhibit incremental growth lines (Lines of incremental growth, **LIG**s). Of these, enamel, as the most biomineralized vertebrate tissue, is more resistant to taphonomic alteration and allows for exceptional preservation of microstructure. Additionally, the presence of LIGs is one of the defining structural features of reptilian enamel (Sander, 1999; Sander, 2000). Also known as striae of Retzius, LIGs have been identified in a variety of extant and extinct amniotes; they have been extensively studied in prismatic mammalian dental enamel (Bromage & Dean, 1985; Dauphin, Jaeger & Osmolska, 1988; Dean & Scandrett, 1996; Dean, 2000), yet also explored in diapsids (Buffetaut et al., 1986; Bocherens, Brinkman & Dauphin, 1994; Line, 2000; Heckert & Miller-Camp, 2013). In mammals, LIGs represent long-period circaseptan amelogenesis (Dean et al., 1993; Dean, 1998; FitzGerald, 1998). However, the molecular mechanisms controlling LIG periodicity in diapsid teeth are incompletely understood. Nevertheless, previous work documents that they are a biologically meaningful indicator of incremental growth of dental tissues (Appenzeller et al., 2005).

Here we use dental histology (including incremental lines in both dentin and enamel) to explore trends in the dental evolution of Therizinosauria, a large-bodied clade of theropod dinosaurs widely regarded to fall on the omnivory/herbivory spectrum (Zanno et al., 2009; Zanno et al., 2016; Zanno, 2010a; Lautenschlager, 2017). Few studies have investigated evolutionary trends in the dental microstructure of theropod dinosaurs within the omnivory/herbivory spectrum, primarily because: (1) most theropod taxa hypothesized to have at least a facultatively herbivorous diet ultimately evolve edentulism, rendering dental comparisons between early and late diverging members difficult to derive, and (2) early diverging species in clades that do retain teeth are generally represented by rare and highly significant specimens that cannot be destructively sampled. Therizinosaurians, by contrast, retain teeth throughout the evolution of the clade, and alterations in tooth morphology and reductions in crown volume in later-diverging members have been identified as ecomorphological correlates of transitions to herbivory (Zanno et al., 2009; Zanno & Makovicky, 2011).

Given the diminutive tooth size documented in several therizinosaurian taxa, we also determine the effect of diet and relative crown volume in dinosaurs. We quantify the relationship between tooth and body size, and assess microstructure and incremental growth in both dentin and enamel of the early-diverging therizinosaurian *Falcarius utahensis*, recovered from a monodominant Lower Cretaceous bonebed in western North America, and *Suzhousaurus megatherioides*, a specialized non-therizinosaurid therizinosauroid from the Lower Cretaceous Xinminpu Group of China. We compare these structures to previously published records of archosaur teeth spanning carnivorous theropods, herbivorous non-theropodan dinosaurs, and crocodilians to assess patterns in enamel and dentin microstructure and crown volume to body mass ratios that may yield insight into the dietary and morphological evolution of the clade. By investigating trends in the microstructure of therizinosaurian dentition, we further efforts to unravel the impact of various tooth growth/replacement strategies on the evolution of key morphological and dietary transitions in Dinosauria.

## Methods and Materials

### Definitions

Enamel microstructure studies were hampered by disagreement on terminology and usage preceding the definitions laid out by Sander (1999), Sander (2000). Tooth terminology follows Sander (1999), Sander (2000). Smith & Dodson (2003) and Hendrickx, Mateus & Araújo (2015a). The schmelzmuster of a tooth is defined as the enamel types present and their spatial distribution in the enamel cap. Parallel crystallite enamel is the simplest and most basal enamel type and is highly conserved in Sauropsida (Sander, 1999; Hwang, 2005; Hwang, 2011). When viewed in longitudinal and transverse section (Erickson, 1996b; Sereno et al., 2007), apatite crystallites run perpendicular to the outer enamel surface (**OES**) and are tightly packed and highly disorganized. Columnar enamel consists of crystallites that form diverging stacks, separated from each other by zones of convergence (Koenigswald & Sander, 1997; Sander, 1999). Additional terminologies differentiating volume-independent tissue deposition/apposition rate from overall tooth growth rate/time were coined for this study, and were adapted from Erickson (1996a), Erickson (1996b) and Line (2000) and are defined as follows: tooth formation time (**TFT**) is the number of days (equivalent to VEL count) present in thin section (“growth rate” *sensu* Erickson, 1996a; Erickson, 1996b). Tooth growth rate (**TGR**) is defined as the crown volume (in ml) divided by the tooth formation time. Dentin deposition rate (**DDR**) is synonymous with mean VEL increment width (*sensu* Erickson, 1996b), and it is the amount of dentin (thickness, in µm) being laid down per day. Similarly, enamel apposition rate (**EAR**) is synonymous with LIG increment width (*sensu* Line, 2000). Unlike TGR and formation time, DDR and EAR are size-independent measures of the production and apposition/deposition of dental tissue that are not affected by variation in VEL count during the ontogeny of individual teeth prior to being shed from the jaw.

The complexity of the schmelzmuster tends to be reflected by the overall complexity of external tooth morphology (Sander, 1999). In some dinosaurian clades such as Hadrosauridae, the schmelzmuster is highly diagnostic, and phylogenetic signals can be read at the suprageneric level (Sander, 1999; Sander, 2000). In Theropoda, there is widespread homoplasy and the schmelzmuster is usually limited to a combination of parallel and columnar enamel, suggesting strong phylogenetic constraints (Stokosa, 2005). However, some functional signals may be present. It is hypothesized that parallel enamel is more resistant to stress from abrasive wear, whereas columnar enamel is structured to withstand torsional stress from bone crushing or prey capture (Stokosa, 2005). Columnar enamel is more often seen in hypercarnivorous theropods such as tyrannosaurids and dromaeosaurids (Sander, 1999; Hwang, 2005; Hwang, 2011; Zanno & Makovicky, 2011).

**Figure 1:**
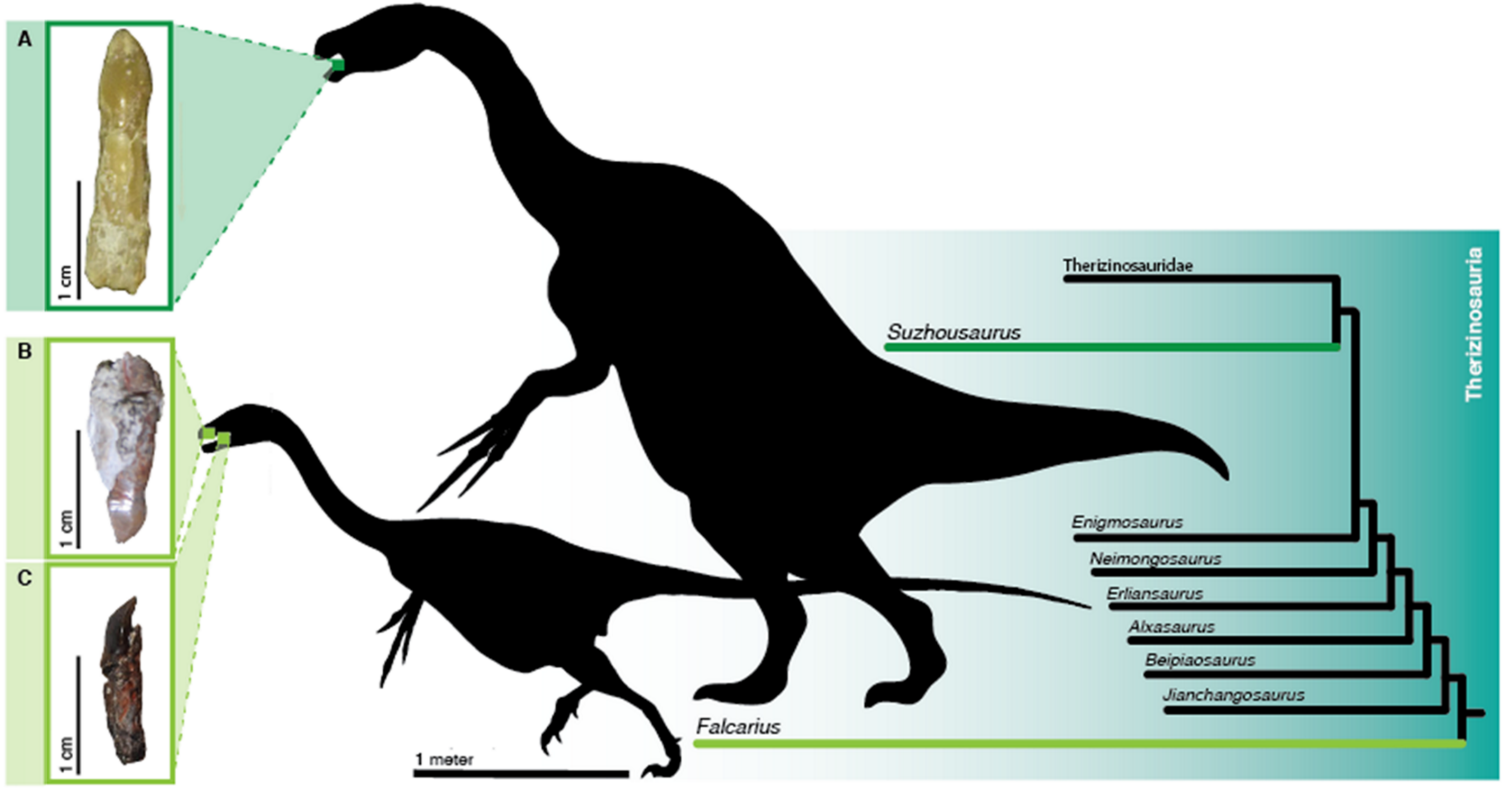
Teeth of sampled therizinosaurians. Isolated teeth of therizinosaurians pre- and post-sectioning. Location in the jaws and phylogenetic positions of therizinosaurians sampled is highlighted.(A) Dentary tooth of *Suzhousaurus megatherioides* in labial view. (B) *Falcarius utahensis* (UMNH VP 22857) maxillary tooth in labial view. (C) Dentary tooth of *Falcarius utahensis* (UMNH VP 15231) in mesial view. Silhouettes of a generalized early diverging and late diverging therizinosauroid modified from skeletal reconstructions published in Kirkland et al. (2005; Greg Paul) and Zanno et al. (2009; Victor Leshyk). Scale bars = 10 µm, 100 µm, 1 cm, and 1 m.

### Sampling methodology

We sampled two isolated teeth (UMNH VP 22857 & 15231, a maxillary tooth and a dentary tooth, respectively) (Figs. 1B and 1C) of *Falcarius utahensis* from the monodominant Lower Cretaceous Crystal Geyser Quarry as well as an uncatalogued tooth referred to *Suzhousaurus megatherioides* collected from the Lower Cretaceous Yujingzi Basin of Gansu, China (Fig. 1A). Following Hwang (2005) and Hwang (2011), we prepared the teeth for microscopy by embedding them in 2-part epoxy and then polished them with fine grain 600–1,200 grit paper. We then cut the teeth in half in longitudinal section using a Buehler Isomet slow-speed saw. We polished the cut faces to remove saw marks, then affixed cut sides to glass petrographic slides using quick-setting epoxy. We made three longitudinal sections each from the *F. utahensis* and *S. megatherioides* teeth. We then polished the sections down to a thickness of approximately 0.1 mm. Using a digital caliper, we confirmed that the slides were of consistent thickness then etched each of the sections for between 30 and 60 s with 1 M hydrochloric acid (Hwang, 2011). The acid was then washed off with tap water. The amount of time for etching varied based on the thickness of the section.

To image the slides, we used a Jeol JSM-6010LA scanning electron microscope set to 20 kV for the *F. utahensis* samples and 5 kV for *S. megatherioides*. We vacuum-coated the slides with approximately 5 nm of Au-Pd to aid in conductance. Au-Pd was later removed with further polishing using 600–1,200 grit paper. We used a Nikon Eclipse Ci-POL petrographic microscope to examine growth lines in dentin. Digital images of VELS were taken and analyzed in ImageJ (Schneider, Rasband & Eliceiri, 2012). Sampling locations are highlighted in Fig. S1. For UMNH VP 22857 and the *Suzhousaurus* tooth, VELs were counted near the base of the crown, where the mineralization front of dentin was roughly parallel with the enamel–dentin junction (**EDJ**). Diagenetic alteration prevented this in UMNH VP 15231, and so a transect closer to the crown apex was used to count VELs in this tooth. Additional images were taken using a VHX-6000 Series Keyence digital microscope.

We calculated total crown volume for UMNH VP 22857 by approximating the tooth as an elliptic solid cylinder and used linear measurements collected in ImageJ. The volume of the pulp cavity was not accounted for due to lack of appropriate measurements. We then adjusted our volume estimate using a direct measurement of volume from UMNH 15231. Volumetric calculations of the remaining teeth were derived from high resolution (0.02 mm) three-dimensional surface scans captured with a Creaform EXAscan handheld scanner using VXelements 3D data acquisition software. Post-processing and generation of 3D models for volumetric calculations was conducted in Geomagic Studio. We calculated enamel volume by subtracting the volume of the tooth without enamel from the total crown volume. To calculate the volume of the tooth without enamel, we took a cylinder uniformly scaled down (geometrically similar but not congruent) from the original cylinder and inset it by the average enamel thickness. Average enamel thickness was calculated by taking the mean of enamel thicknesses, measured normal to the enamel-dentin junction (**EDJ**), from a transect of 15 regularly spaced points running apicobasally (7 from each side and one from near the tip) using ImageJ. Further linear measurements of crown height and total tooth length were taken for additional *F. utahensis* dentary teeth (UMNH VP 14527 and UMNH VP 15259) preserved *in situ* in the mandible (Fig. S2). Crown volumes for other, primarily carnivorous, theropods were also approximated as elliptic cylinders and were calculated using data from Hendrickx, Mateus & Araújo (2015b).

### Dietary classifications and body mass estimates

Taxa were binned as being (1) primarily carnivores, (2) primarily herbivores. Therizinosaurians were considered a special case and were analyzed separately. We compensated for uncertain dietary classification in *Troodon* and *Eoraptor* by running multiple analyses classifying the taxa as first carnivores, then herbivores, and lastly by dropping them from our dataset. Body masses were taken from Benson et al. (2014). In instances where multiple mass estimates were available for a given taxon, the largest value was used (see Table 1).

### Statistical analysis

We used a two-sided homoscedastic *t*-test to compare DDR in therizinosaurs to that of other archosaurs. To compare crown volume:body mass ratios in Therizinosauria, we employed a Kruskal–Wallis test with permutation *p*-value. We then performed a Kruskal–Wallis multiple comparisons test. We used a Spearman’s rank correlation permutation test to examine the relationship between DDR and replacement rate. To target potential confounding factors, we compared DDR between matching pairs of juveniles and adults of the same species using 2-sided paired *t*-test. Published data from Erickson (1996b), D’Emic and colleagues (1993), Benson and colleagues (2014), Hendrickx, Mateus & Araújo (2015b), and Zanno and colleagues (2016). All tests were conducted in RStudio Team (2009–2017) 1.0.153. Permutation tests used 5,000 replicates.

**Table 1:** Crown volume and body mass in archosaurs. Taxa are categorized by diet and growth stage: Red, Primarily carnivorous; Green, Primarily herbivorous; and Gold, Unknown/omnivore. Lighter colors represent juvenile/sub-adult specimens. Therizinosaurians are in bold.

Taxon	*n*	Mean crown volume, mL	Body mass, kg	CV:BM ratio, mL/kg	Growth stage	Source
*Acrocanthosaurus*	34	16.23	3.50E+3	4.64E−03	NA	^f^
*Afrovenator*	1	11.97	1.00E+3	1.20E−02	?	^f^
Albertosaur	1	18	1.30E+3	1.38E−02	Adult	^b^
*Albertosaurus*	1	8.26	2.00E+3	4.13E−03	?	^f^
*Alioramus*	15	1.1	2.80E+2	3.91E−03	NA	^f^
*Alligator*	3	0.12	5.91	2.03E−02	Juvenile	^a^
*Allosaurus*	31	3.62	2.50E+3	1.45E−03	NA	^f^
*Aucasaurus*	2	2.13	8.50E+2	2.51E−03	NA	^f^
*Australovenator*	6	0.48	3.10E+2	1.55E−03	NA	^f^
*Bambiraptor*	10	0.01	4.00	2.62E−03	NA	^f^
*Carcharodontosaurus*	14	31.6	3.00E+3	1.05E−02	NA	^f^
*Carnotaurus*	4	2.05	1.60E+3	1.28E−03	NA	^f^
*Ceratosaurus*	16	6.31	5.00E+2	1.26E−02	NA	^f^
*Coelophysis*	20	0.02	9.00	2.31E−03	NA	^f^
*Daspletosaurus*	15	12.28	6.10E+2	2.01E−02	NA	^f^
*Deinonychus*	1	0.2	1.00E+2	2.00E−03	Adult	^b^
*Deinonychus*	12	0.12	9.70E+1	1.27E−03	NA	^f^
*Dilophosaurus*	4	3.02	2.90E+2	1.04E−02	NA	^f^
*Erectopus*	3	4.55	3.00E+2	1.52E−02	NA	^f^
*Fukuiraptor*	1	2.19	2.50E+2	8.77E−03	?	^f^
*Giganotosaurus*	7	28.28	6.10E+3	4.64E−03	NA	^f^
*Gorgosaurus*	17	4.8	2.50E+3	1.92E−03	NA	^f^
*Leidyosuchus*	1	1.2	2.15E+2	5.57E−03	?	^b^
*Liliensternus*	7	0.12	8.40E+1	1.45E−03	NA	^f^
*Majungasaurus*	1	1.58	1.60E+3	9.86E−04	?	^f^
*Mapusaurus*	7	11.4	4.10E+3	2.78E−03	NA	^f^
*Masiakasaurus*	22	0.05	2.00E+1	2.55E−03	NA	^f^
*Megalosaurus*	13	5.1	1.40E+3	3.64E−03	NA	^f^
*Neovenator*	3	2.25	1.00E+3	2.25E−03	NA	^f^
*Raptorex*	17	0.26	4.40E+1	5.94E−03	NA	^f^
*Saurornitholestes*	117	0.05	1.80E+1	2.99E−03	NA	^f^
*Skorpiovenator*	2	2.57	1.20E+3	2.14E−03	NA	^f^
*Suchomimus*	20	4.55	2.90E+3	1.57E−03	NA	^f^
*Torvosaurus*	4	31.57	2.40E+3	1.32E−02	NA	^f^
*Tyrannosaurus*	1	138	7.70E+3	1.79E−02	Adult	^b^
*Tyrannosaurus*	1	15.5	6.16E+3	2.52E−03	Sub-adult	^b^
*Tyrannosaurus*	1	1.8	1.00E+3	1.80E−03	Juvenile	^b^
*Tyrannosaurus*	131	41.26	7.70E+3	5.36E−03	NA	^f^
*Velociraptor*	20	0.03	1.20E+1	2.47E−03	NA	^f^
***Beipiaosaurus***	**1**	**0.02**	**4.72E + 1**	**3.57E–04**	**?**	^c^
*Camarasaurus*	?	15.7	4.70E+4	3.34E−04	NA	^e^
*Diplodocus*	?	1.51	1.30E+4	1.16E−04	NA	^e^
*Edmontonia*	1	0.2	3.00E+3	6.67E−05	Adult	^b^
*Edmontosaurus*	2	2	3.00E+3	6.67E−04	Adult	^b^
*Edmontosaurus*	3	0.43	7.00E+2	6.14E−04	Juvenile	^b^
*Maiasaura*	1	1.9	3.60E+3	5.28E−04	Adult	^b^
*Prosaurolophus*	3	2	4.50E+3	4.44E−04	Adult	^b^
***Segnosaurus***	**1**	**0.28**	**2.23E + 3**	**1.25E–04**	**?**	^d^
***Suzhousaurus***	**1**	**0.05**	**3.10E+3**	**1.76E–05**	**?**	^c^
*Triceratops*	2	2.65	9.00E+3	2.94E−04	Adult	^b^
***Falcarius (UMNH VP 15231)***	**1**	**0.02**	**1.00E + 2**	**2.00E–04**	**Juvenile?**	^c^
***Falcarius (UMNH VP 22857)***	**1**	**0.03**	**1.00E + 2**	**3.41E–04**	**Juvenile?**	^c^
*Eoraptor*	26	0.01	1.70E+1	8.51E−04	NA	^f^
*Troodon*	2	0.04	5.00E+1	8.00E−04	Adult	^b^
*Troodon*	35	0.04	4.70E+1	9.30E−04	NA	^f^

**Notes.**

a Erickson (1996a).

b Erickson (1996b).

c This paper.

d Zanno et al. (2016).

e D’Emic et al. (2013).

f Hendrickx, Mateus & Araújo (2015b).

## Results

Therizinosaurian teeth are folidont (*sensu* Hendrickx, Mateus & Araújo, 2015a) in form and are generally diminutive (Fig. S2) (but see Zanno et al., 2016), measuring no more than 9.0 mm in crown height and 3.8 mm in crown base length in our sample. Minute denticles are present on the distal carinae of teeth from both taxa. Folding of the carinae, seen in the therizinosaurid *Segnosaurus galbinensis* (Zanno et al., 2016), is not present in *F. utahensis* or *S. megatherioides*.

### Falcarius utahensis

#### Gross description

Pre-sectioned total tooth lengths (crown and preserved root) were 14.752 and 13.301 mm; crown heights were 4.265 and 6.21 mm, respectively. This yields a mean root/crown height ratio of 2.78. Crown base widths were 2.184 and 2.500 mm and crown base lengths were 3.538 and 3.620 mm. There were no signs of wear on either tooth. The dentary tooth (UMNH 15231) was labiolingually constricted at the cervix (crown base ratio 0.62), and the crown was slightly distally and lingually recurved and ‘D’-shaped in cross-section. There was little crown ornamentation, but a lingual depression was present. The tip of the crown apex (*sensu* Hendrickx, Mateus & Araújo, 2015a) was missing.

The maxillary tooth (UMNH 22857) had a larger crown base ratio (0.69), as it was more constricted mesiodistally than labiolingually at the cervix. It was more strongly lingually recurved than the dentary tooth but less distally inclined. Its cross section was more lenticulate. It was mesiodistally expanded (mid-crown ratio 0.44) and bore a small longitudinal ridge (*sensu* Hendrickx, Mateus & Araújo, 2015a) on the lingual aspect.

#### Dental histology

**Figure 2:**
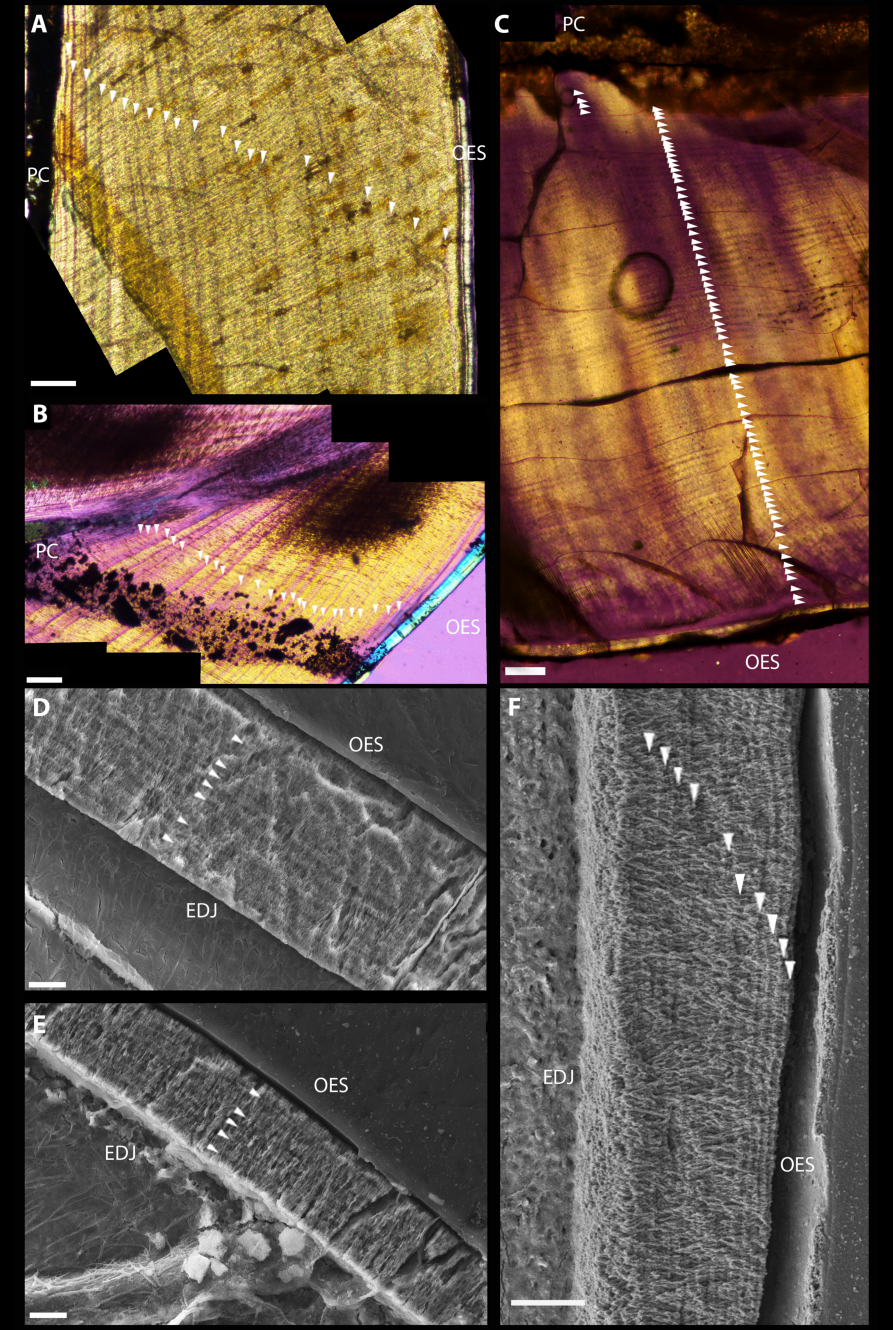
Dental microstructure in Therizinosauria. (A–C) Polarized light microscopy of longitudinally sectioned teeth. (A) UMNH VP 22857. Dentin preserves 38 VELs with a mean increment width of 13.54 µm. (B) UMNH VP 15231. Dental histology reveals 44 VELs with mean increment width 18.12. (C) *Suzhousaurus* tooth. 79 VELs with a mean increment width of 16.0 µm were visible in section. (D–F) Scanning Electron Microscopy images of longitudinally sectioned teeth. (D) Dental enamel of UMNH VP 22857. An average of 6.00 ± 1.53 LIGs were identified, with mean increment width 5.35 µm. (E) The enamel of UMNH VP 15231 preserves 5.00 ± 0.71 LIGs with mean increment width 3.495. Schmelzmuster of both *Falcarius* teeth consisted of parallel crystallite enamel only. (F) Schmelzmuster of *Suzhousaurus* enamel consists of slightly divergent parallel crystallite enamel. An average of 15.67 ± 5.70 LIGs were counted with a mean increment width of 2.06 µm. Incremental growth lines indicated with white arrows. Scale bars = 100 µm (A–C) and 10 µm (D–F). Abbreviations: EDJ, enamel–dentin junction; OES, outer enamel surface; PC, pulp cavity.

Polarized light microscopy revealed 38 VELs in UMNH VP 22857 (Fig. 2A) and 44 in UMNH 15231 (Fig. 2B). The mean increment width was 15.8 µm. Total crown volume (**TCV**) was 20.00 mm^3^ for UMNH 22857 and 34.10 mm^3^ for UMNH 15231. Maximum enamel thickness was 50 µm; average thickness was approximately 33.4 µm. Enamel on the occlusal and labial sides was twice as thick as the lingual aspect. Miniscule serrations, visible under a hand lens, were present on the distal carina. Calculated enamel volume averaged 0.70 mm^3^, making up 2.58% of TCV. The schmelzmuster of both was almost exclusively comprised of simple parallel crystallite enamel with no other enamel types present; however, a basal unit layer is discernible in UMNH VP 15231 (Fig. 2E). Clear, regularly-spaced LIGs (Figs. 2D–2F) were distributed from the EDJ to the OES. An average of 6.00 ± 1.53 LIGs were counted for UMNH VP 22857 (Fig. 2D) and 5.00 ± 0.71 LIGs for UMNH VP 15231 (Fig. 2E). Mean LIG increment width was 4.29 µm, which is comparable to that of a theropod tooth examined by Line (2000).

### Suzhousaurus megatherioides

#### Gross description

The tooth was conodont in form and more symmetrical (both mesiodistally and labiolingually) than the *F. utahensis* teeth, with only slight constriction (crown base width approximately equal to mid-crown width). A small lingual depression was present with a narrow longitudinal ridge flanked by distal and mesial longitudinal ridges. Its cross-section was subcircular. Pre-sectioned total tooth length was 2.10 cm (root plus crown), with a crown 0.881 cm in height yielding a root/crown height ratio of 2.38. Crown base length was 0.38 cm and crown base width was 0.27 cm. It was relatively more slender, with higher crown height ratio. Unlike in *F. utahensis* teeth, a shallow wear facet, only visible under hand lens, was present on the labial side of the tooth, near to the crown apex. Even prior to sectioning, the tooth was nearly translucent.

#### Dental histology

After polishing the sections down to 0.1 mm thick, the crown was completely transparent, and only the pulp cavity remained opaque. Microscopic denticles, not observed upon initial survey of external morphology, were visible in section on the lingual aspect immediately adjacent to the crown apex. Under polarized light microscopy, 79 VELs with a mean increment width of 16.0 µm were visible in section (Fig. 2C). Raw DDR data and VEL counts for *S. megatherioides* and *F. utahensis* are compiled in Table S1. Maximum enamel thickness was approximately 120 µm near the tip of the crown on the lingual side, and 90 µm on the labial side. The average thickness was approximately 40 µm. TCV was 54.69 mm^3^ and total enamel volume was 2.94 mm^3^ (5.37% TCV). The schmelzmuster also consisted of parallel crystallite enamel, but unlike *F. utahensis* the crystallites were slightly divergent. *S. megatherioides* teeth also possess LIGs parallel with the boundary of the OES, yet these were more numerous than *F. utahensis*. An average of 15.67 ± 5.70 LIGs were counted with a mean increment width of 2.06 µm (Fig. 2F). Enamel volume and apposition rate raw data is compiled in Table S2.

#### Statistical testing

**Figure 3:**
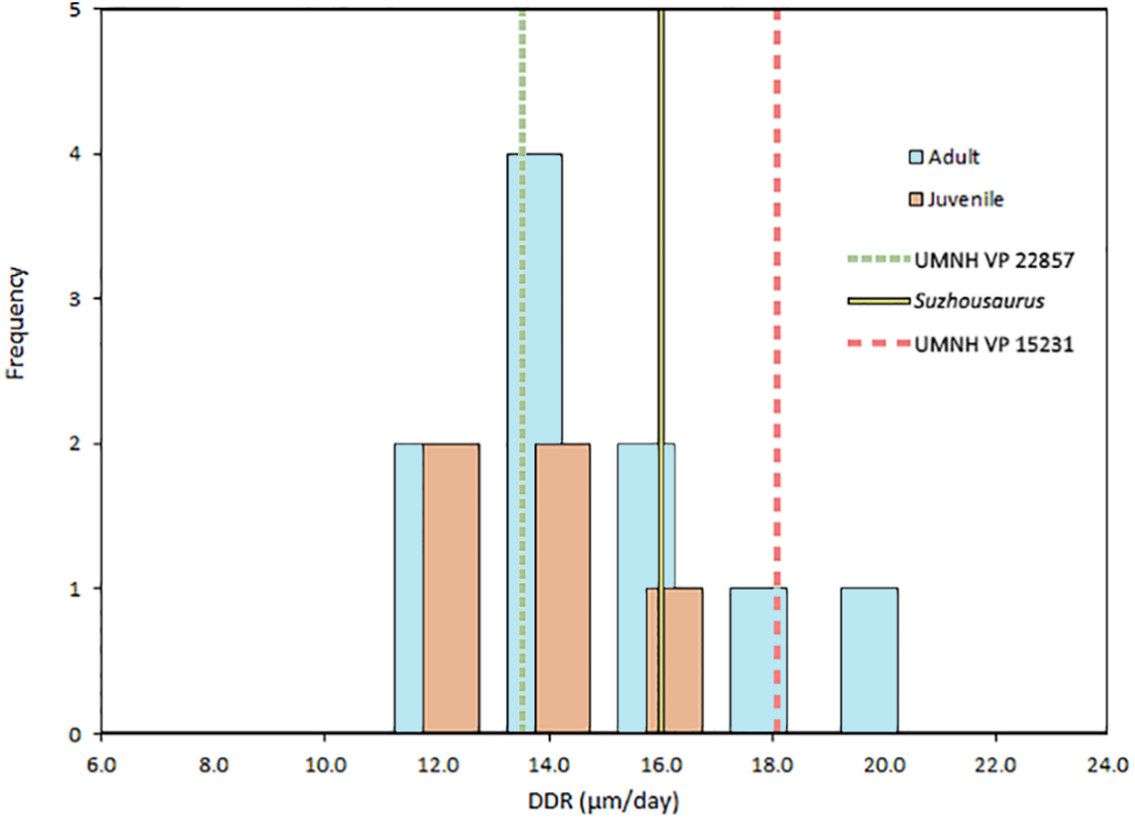
Dentin deposition rates in Archosauria. Dentin deposition rate for archosaurs falls in a narrow range between 10 and 20 µm/day. Mean DDR in therizinosaurians is 15.89 µm/day, not significantly different (*p* = 0.323) from other Archosauria (14.225 µm/day), but much higher than that of mammals, which average around 3 µm/day (Dean, 1998). Mean DDR was 13.87 µm/day for juveniles and 14.42 µm/day for adults. DDR data from Erickson (1996b), and D’Emic et al. (2013) and is compiled in Table S3.

**Figure 4:**
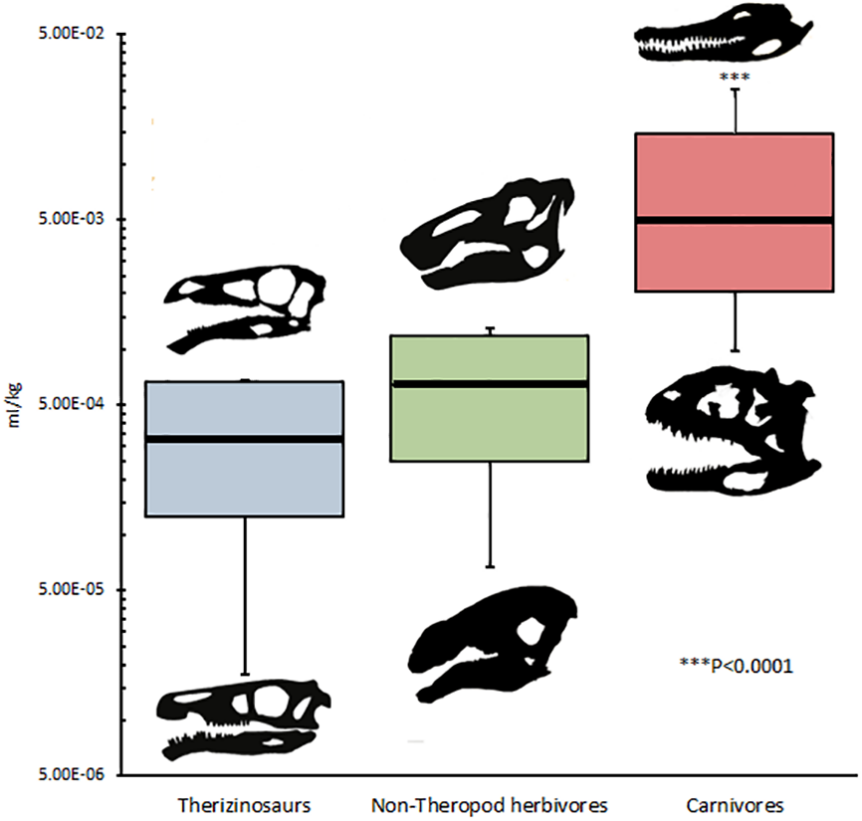
Crown volume to body mass ratios in archosaurs. Mean CV:BM ratio for Therizinosauria was 1.84E–4 ml/kg; mean CV:BM ratio for non-theropod herbivores is 3.83E–4 ml/kg; mean CV:BM ratio for carnivores is 6.35E–3 ml/kg, significantly (*p* < 0.0001) greater than that of non-carnivores, which are statistically indistinguishable from one another. Skull silhouettes represent taxa occupying local maxima for each diet category. Body masses taken from Benson et al. (2014). Crown volume was calculated by approximating teeth as elliptic cylinders with major axis equivalent to crown base length, minor axis crown base width, and crown height (*sensu* Hendrickx, Mateus & Araújo, 2015a). Published tooth volumes and measurements are from Erickson (1996b), Hendrickx, Mateus & Araújo (2015b), and Zanno et al. (2016). See Table 1 for complete list of taxa and references used in this analysis. Silhouettes generated in Paint.NET by KB using public domain images.

DDR in therizinosaurs is not significantly different (*p* = 0.323) from that of other archosaurs and was not significantly correlated with replacement rate (*p* = 0.3356). DDR in adults was an average of 0.54 µm/day greater than in juveniles, however this difference was not significant (*p* = 0.55) (Fig. 3). Mean crown volume to body mass ratios for therizinosaurs and other herbivorous dinosaurs were 1.84E–4 and 3.83E–4 ml per kg, respectively, whereas CV:BM ratio for hypercarnivorous archosaurs (5.60E–3 ml/kg) was significantly higher (*p* < 0.0001) than that of therizinosaurs and non-theropod herbivores (Fig. 4). Additional DDR raw data as well as CV and BM data are compiled in Table S3.

## Discussion

**Figure 5:**
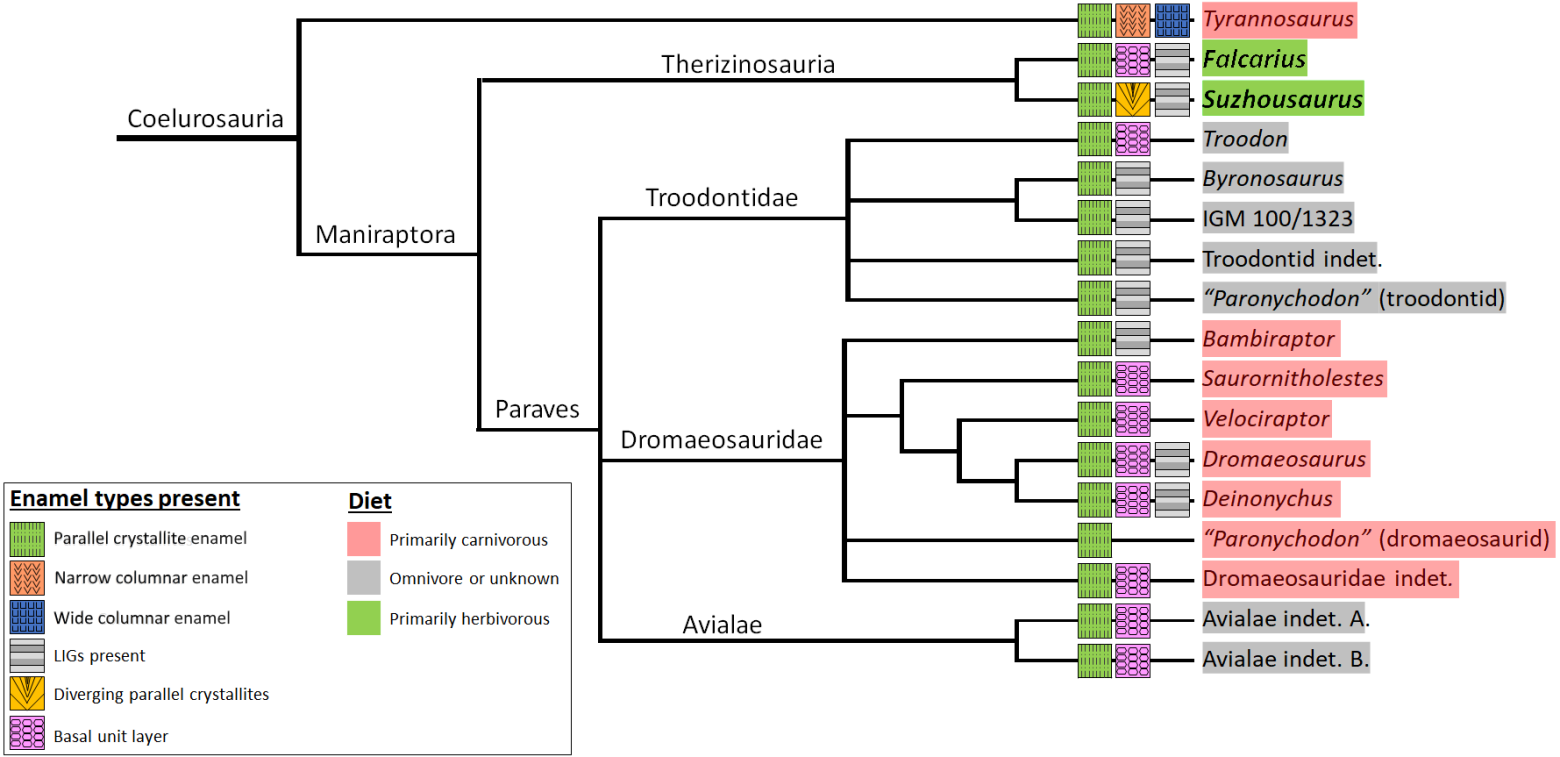
Diet and schmelzmuster in coelurosauria. Homoplasy is widespread, and parallel crystallite enamel is commonplace across the clade. Schmelzmuster does not appear to strongly reflect diet or phylogenetic position within the clade. Therizinosaurians possess simplistic enamel with no columnar units, and with basal unit layer only detectable in UMNH VP 15231. Enamel crystallites in *Suzhousaurus* are slightly divergent. Data from Hwang (2011). Phylogeny from Cau, Brougham & Naish (2015).

Although more research has focused on growth rates based on VEL counts (Erickson, 1996b; Sereno et al., 2007; D’Emic et al., 2013; García & Zurriaguz, 2016), we chose to examine both dentin and enamel due to the resilience of enamel microstructure in the fossil record (Sander, 1999). The schmelzmuster of Therizinosauria is remarkably similar to other closely related theropod subclades (Fig. 5), despite dietary differences, supporting previous hypotheses of strong phylogenetic constraints on the evolution of enamel microstructure (Hwang, 2005). Therizinosaurians, which are widely regarded to fall within the omnivory/herbivory spectrum (Barrett, 2005; Zanno et al., 2009), show the same parallel crystallite enamel found in hypercarnivorous dromaeosaurids and thus we observe no strong dietary signal amongst those maniraptorans whose dental histology has been categorized (Stokosa, 2005; Zanno & Makovicky, 2011; Hwang, 2011) and can reject the hypothesis that dietary differences in Therizinosauria are evident in enamel microstructure. In a functional context, simple parallel crystallite enamel is regarded the least specialized enamel type (Sander, 1999; Sander, 2000) and is plesiomorphic for Maniraptora. Thus, the lack of microstructural specialization in the two therizinosaurians sampled here suggests that most members of the clade were not heavily reliant on oral food processing and did not experience selective pressure to modify dental microstructure to cope with changing tooth function. This interpretation fits with the simplistic gross dental morphology of nearly all known therizinosaurians. One notable exception is the therizinosaurid *Segnosaurus galbinensis*, which can be expected to vary from patterns observed given a hypothesized increase in oral processing based on specialized dental traits (Zanno et al., 2016) and the strongest potential bite force among those therizinosaurians that can be confidently modeled (i.e., preserve post-dentary mandibular elements) (Lautenschlager, 2017). The slightly divergent parallel enamel of the late-diverging non-therizinosaurid therizinosauroid *S. megatherioides* may reflect more orderly enamel production, and potentially increasing functional specializations bridging the simplistic teeth of *F*. *utahensis* and the specialized teeth and higher bite force simulations of the therizinosaurid *S*. *galbinensis*; however we caution against ascribing too much significance to a potential increase in enamel organization coinciding with increasing skeletal adaptations for herbivory in the clade given the dearth of microstructure data currently available.

Analysis of incremental growth lines provides a more informative picture of the evolution of therizinosaurian dental tissues. We estimate a 79–80 day tooth formation time for the buccal dentition of *S*. *megatherioides*; *Falcarius utahensis* teeth form in approximately half that duration (38–45 days) and comprise approximately half the crown volume of *S*. *megatherioides* teeth, which is consistent with the conservation of average increment width (**DDR**) we observe in both taxa (between 15.8–16 µm/day). Not only was DDR statistically indistinguishable between the taxa sampled in our study, it was not significantly different from dentin deposition rates calculated for more distantly-related taxa. Despite dietary differences, the average DDR across Dinosauria resides within a narrow band between 10–20 µm per day (Fig. 3). This does not appear to be strongly influenced by sampling location on the tooth; although the position of the transect along which VEL increment widths were calculated varied between our two *F. utahensis* specimens, they both fell within the range expected for dinosaurian dentition. This data supports previous findings regarding the action of physiological and/or structural limitations on the rate of dental tissue growth and deposition in both embryonic and post-hatching archosaurian dentition (Erickson, 1996b; Erickson et al., 2017). Even megaherbivorous taxa such as *Triceratops* and *Edmontosaurus* that evolved dental batteries with elevated rates of tooth replacement to increase oral processing capacity are limited to average dentin deposition rates of 15.8 and 19.8 µm per day, respectively (Erickson, 1996b). Additionally, our data shows no correlation between DDR and replacement rate, suggesting that these taxa increased replacement rates despite constraints on the rate of tissue production. This provides methodological support for previous efforts to non-destructively estimate VEL count (and ultimately, replacement rates) by dividing cross-sectional dentin thickness (calculated from CT scans) by a given increment width (D’Emic et al., 2013).

The pattern observed in therizinosaurian dentin in our sample is not repeated in enamel microstructure. LIGs were denser and more numerous in *Suzhousaurus* than in *Falcarius*. Assuming LIG periodicity is conserved between these taxa (Appenzeller et al., 2005), we recover a pronounced decrease in enamel apposition rate (52%) in the more specialized therizinosaurian *S. megatherioides*, from the condition observed in the early-diverging taxon *F*. *utahensis*. Decrease in enamel apposition rate in specialized therizinosaurians contrasts with increasing relative thickness of the enamel when normalized against crown volume, from 2.58% in *F*. *utahensis* to more than double that value (5.37%) in *S*. *megatherioides*. Given the decreasing enamel apposition rate suggested by our data, greater enamel to crown volume ratio in *S*. *megatherioides* would necessitate significantly longer enamel formation times. Although the total volume of enamel on the teeth of *S*. *megatherioides* took, on average, three times as long to deposit as that of *F*. *utahensis* dentition (based on LIG count), this does not adequately account for twice the relative volume of enamel deposition at half the rate of apposition and suggests that there are other, as of yet unidentified, variables factoring into these values, including the possibility that LIG periodicity is not conserved. Direct comparisons between the distribution of LIGs in therizinosaurians and other dinosaurian clades on the omnivory/herbivory spectrum may help uncover these factors, yet currently cannot be made because growth lines in enamel are undersampled in Dinosauria.

Analysis of relative tooth size provides a more conclusive dietary signal. In terms of both absolute values and as a function of estimated body mass (Table 1), Therizinosaurians possess the smallest teeth in our dataset. Crown volume to mass ratios in Therizinosauria more closely resemble those of non-theropod herbivorous dinosaurs (Fig. 4) than those of hypercarnivorous coelurosaurians such as *Tyrannosaurus* and *Deinonychus* or the potentially omnivorous taxon *Troodon*, which have crown volume to body mass ratios that are one to two orders of magnitude greater than that of the therizinosaurians sampled. This finding supports the presence of a dietary signal in quantifications of tooth volume to mass ratios in dinosaurs, and provides an independent line of evidence for contrasting dietary strategies between therizinosaurians (predominant herbivores) and carnivorous coelurosaurians, as well as coelurosaurs of ambiguous diet (e.g., *Troodon*). Additional data from other coelurosaurians, particularly small-bodied paravians, may increase the degree of confidence that can be applied to this finding, yet crown volumes of teeth that can confidently be assigned to taxa where body mass estimation is possible are undersampled in this clade and others (Hendrickx, Mateus & Araújo, 2015a; Hendrickx, Mateus & Araújo, 2015b).

Erickson (1996b) observed that VEL increment widths tend to increase with ontogeny, so growth stage must be considered a potential confounding variable in our study, particularly given the small sample sizes we were able to obtain. Although we cannot determine the ontogenetic stage of the individual *Falcarius* teeth sampled because they were found as isolated specimens in a bonebed, the majority of *Falcarius utahensis* materials recovered from the site to date derive from individuals that were actively growing at the time of death (Zanno & Erickson, 2006); therefore, UMNH VP 22857 & 15231 likely derive from a similar growth stage (Zanno, Kirkland & Herzog, 2014). If ontogeny has an effect on VEL widths and if the *S. megatherioides* tooth belonged to a skeletally mature individual, this might bias our results. To determine if ontogeny was a statistically significant factor in VEL increment widths, we applied a two-sample random permutation test to Erickson’s (1996b) dataset. We found that DDR was generally higher in skeletally mature as opposed to actively growing individuals; however, we are unable to find statistically significant support for this difference, and suggest the trend may be species-variable.

Enamel apposition continues until tooth eruption (Zahradnicek, Horacek & Tucker, 2012) therefore, the developmental stage reached by the individual teeth in our sample could impact LIG count. Because none of the teeth in our sample were recovered within the dentigerous cranial elements, and functional *Falcarius* teeth do not exhibit wear facets (Zanno, 2010b), we assessed whether our sampled teeth were likely to have been functional (erupted tooth crowns) via comparative measurements and CT imaging of *Falcarius* teeth preserved within the mandible. Computed tomographic images of dentigerous dentaries indicate that *F. utahensis* crowns are not completely formed in the maxilla or dentary until they have begun erupting from the alveolus (Fig. S3), and in all teeth bearing a root of equal length to the crown height, the crown is entirely erupted from the alveolus (see Lautenschlager et al., 2014). Our sampled *Falcarius* teeth bear root/crown length ratios ranging between (2.38–2.78), therefore, we feel confident that LIG deposition had ceased in these teeth. No dentigerous elements are known for *S. megatherioides*; however, given that the observed pattern of tooth development/eruption stage in *Falcarius* is also known for the late diverging taxon *Erlikosaurus andrewsi* (Lautenschlager et al., 2014), it is not unreasonable to suggest this pattern characterizes therizinosauroids generally, and that we can reasonably infer that the *S. megatherioides* tooth sampled represented a functional tooth.

In contrast to enamel apposition, which ceases following tooth eruption, dentin deposition continues until teeth are shed (Edmund, 1969). The therizinosauroid teeth in our sample possessed fully developed roots and were therefore functional, not shed teeth. We would therefore expect our tooth formation times (TFT) to represent minimum values for sampled teeth as VEL count would be expected to increase. VEL count is not used to calculate DDR, which is based on increment width, therefore the formation stage of sampled teeth would not affect the majority of results discussed here.

## Conclusion

We repurpose and more explicitly define existing terminology describing incremental growth of dental tissues in dinosaurs. When analyzing tooth growth, it is vital to differentiate volume-independent measures of deposition/apposition rates and overall tooth growth rates/times. Despite occupying opposite ends of the dietary spectrum from the majority of theropod dinosaurs, therizinosaurian teeth do not exhibit histological specializations to diet as seen in other herbivorous dinosaurs. Rather, their dental microstructure is composed of simple parallel crystallite enamel, supporting previous suggestions of reduced reliance on oral processing in the clade equally with hypotheses of a strong phylogenetic signal in enamel microstructure in dinosaurs. Although sample size is limited, we document strong reduction in enamel apposition rate (52%) and approximate doubling in their enamel thickness (from 2.58% to 5.37%) between early-diverging therizinosaurians and large-bodied, highly specialized therizinosauroids. Specializations in therizinosaurian enamel during the evolutionary history may be linked to increasing herbivory in the clade, yet assessment of this hypothesis must await broader taxonomic sampling and increased ontogenetic controls.

Variation in enamel growth and histology between early and later diverging therizinosaurians contrasts with a uniform dental deposition rate. Taking crown volume into account, we find further support for the hypothesis that dentin deposition rate is restricted to a narrow range of values related to physiological limitations on the rate of dental tissue production. Therizinosaurians are regarded to fall on the omnivory/herbivory spectrum and have unusually diminutive teeth. We calculate raw crown volumes and crown volume relative to body mass to be the smallest value in our archosaurian sample. As opposed to the weak dietary signal observed in tooth microstructure, crown volume to body mass ratios of therizinosaurian teeth fall closest to the range of values documented for non-theropodan herbivorous dinosaurs and contrast strongly with those of hypercarnivorous taxa. Thus, crown volume estimates controlling for mass may provide a novel independently quantifiable metric for contrasting hypotheses of diet in Dinosauria.

##  Supplemental Information

10.7717/peerj.4129/supp-1 Supplemental Information 1 Location of transects on sectioned teeth Digital microscopy images of therizinosaurian teeth in longitudinal thin section. (A) UMNH VP 22857 maxillary tooth. (B) UMNH VP 15231 dentary tooth. (C) *Suzhousaurus* tooth. Red boxes indicate location of transects sampled in Figs. 2A–2C. Scale bar represents 500 µm.

10.7717/peerj.4129/supp-2 Supplemental Information 2 *In situ Falcarius utahensis* dentary teeth UMNH VP 14527 (A–C) and UMNH VP 15259 (D, E) in labial (A, D), lingual (B, E) , and occlusal (C) views. Scale bar represents 1 mm.

10.7717/peerj.4129/supp-3 Supplemental Information 3 Computed tomographic images of *Falcarius utahensis* dentary teeth (A) UMNH VP 14527 in transverse (labiolingual) cross-section. (B) UMNH VP 14529 in transverse cross-section. Crowns are not completely formed until they have begun erupting from the alveolus. Root lengths that equal or exceed crown height are indicative of fully erupted functional teeth.

10.7717/peerj.4129/supp-4 Supplemental Information 4 von Ebner increment widths in Therinosauria Summary: Summary statistics for von Ebner line increment widths in thin sections of *F. utahensis* and *S. megatherioides* teeth. Raw: measured increment widths from histological thin sections of therizinosaurian teeth and distribution of thin VELs from EDJ to pulp cavity. Digital microscope images were taken using a Nikon Instruments DS-Fi2 microscope camera and analyzed in ImageJ.

10.7717/peerj.4129/supp-5 Supplemental Information 5 Enamel apposition rates and enamel/crown volumes in therizinosauria Enamel Apposition Rates: calculated EAR in *F. utahensis* and *S. megatherioides*. EAR decreases by 52% in *Suzhousaurus* and LIG count is 85% greater than in *Falcarius*. However, enamel volume is 2.79 percentage points greater in the more derived taxon. Crown and Enamel Volumes: Calculated Crown volume and enamel percentage in sampled therizinosaurians. Teeth were approximated as elliptic cylinders with semi-major axis = crown base length and semi-minor axis = crown base width. Enamel volume was estimated as a hollow elliptic cylinder with thickness = average enamel thickness.

10.7717/peerj.4129/supp-6 Supplemental Information 6 Crown volume, body mass, and dental microstructure raw data from sampled taxa Raw data from this study as well as Erickson (1996b) and D’Emic et al., 2013 on crown volumes, body masses, and dental microstructure in Archosauria. Body mass data from Benson et al., 2014. DDR vs. Replacement: No significant correlation was found between deposition rate and replacement rate (measured in teeth replaced per day, *contra* Erickson, 1996b). DDR by diet: Comparison of dentin deposition rates by diet category in archosaurs. DDR Juvenile vs. Adult: Comparison of dentin deposition rates between growth stage categories.

## Additional Information and Declarations

### Data Availability

The following information was supplied regarding data availability: The raw data has been provided as Supplemental Files.
