# Supplementary figures and images for: Incremental growth of therizinosaurian dental tissues: implications for dietary transitions in Theropoda
Source: PeerJ. 2017 Dec 11;5:e4129. doi: 10.7717/peerj.4129 (PMC5729821; doi:10.7717/peerj.4129)

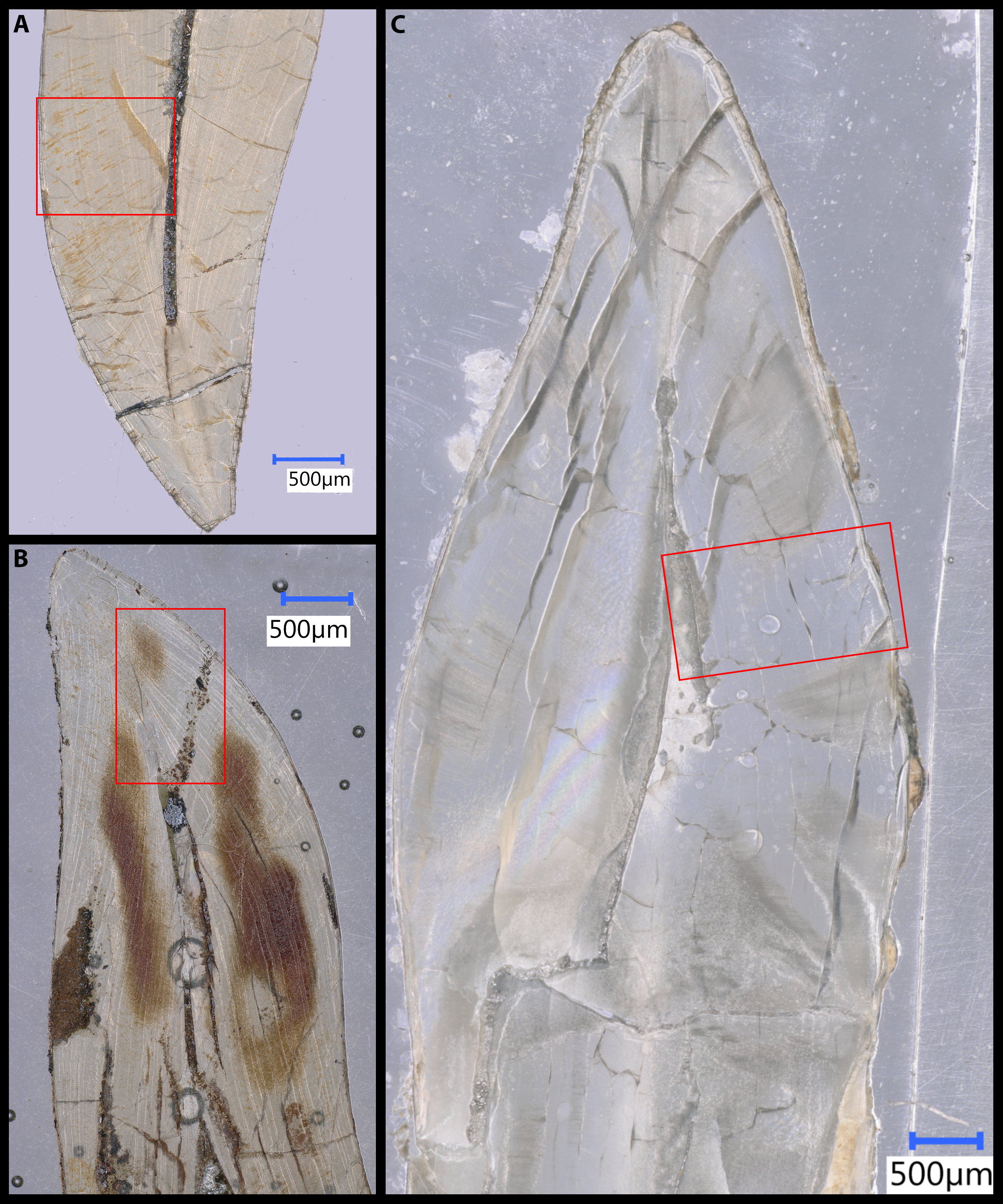

Supplement: Supplemental Information 1 — Digital microscopy images of therizinosaurian teeth in longitudinal thin section. (A) UMNH VP 22857 maxillary tooth. (B) UMNH VP 15231 dentary tooth. (C) Suzhousaurus tooth. Red boxes indicate location of transects sampled in Figs. 2A–2C. Scale bar represents 500 µm. [file peerj-05-4129-s001.png]

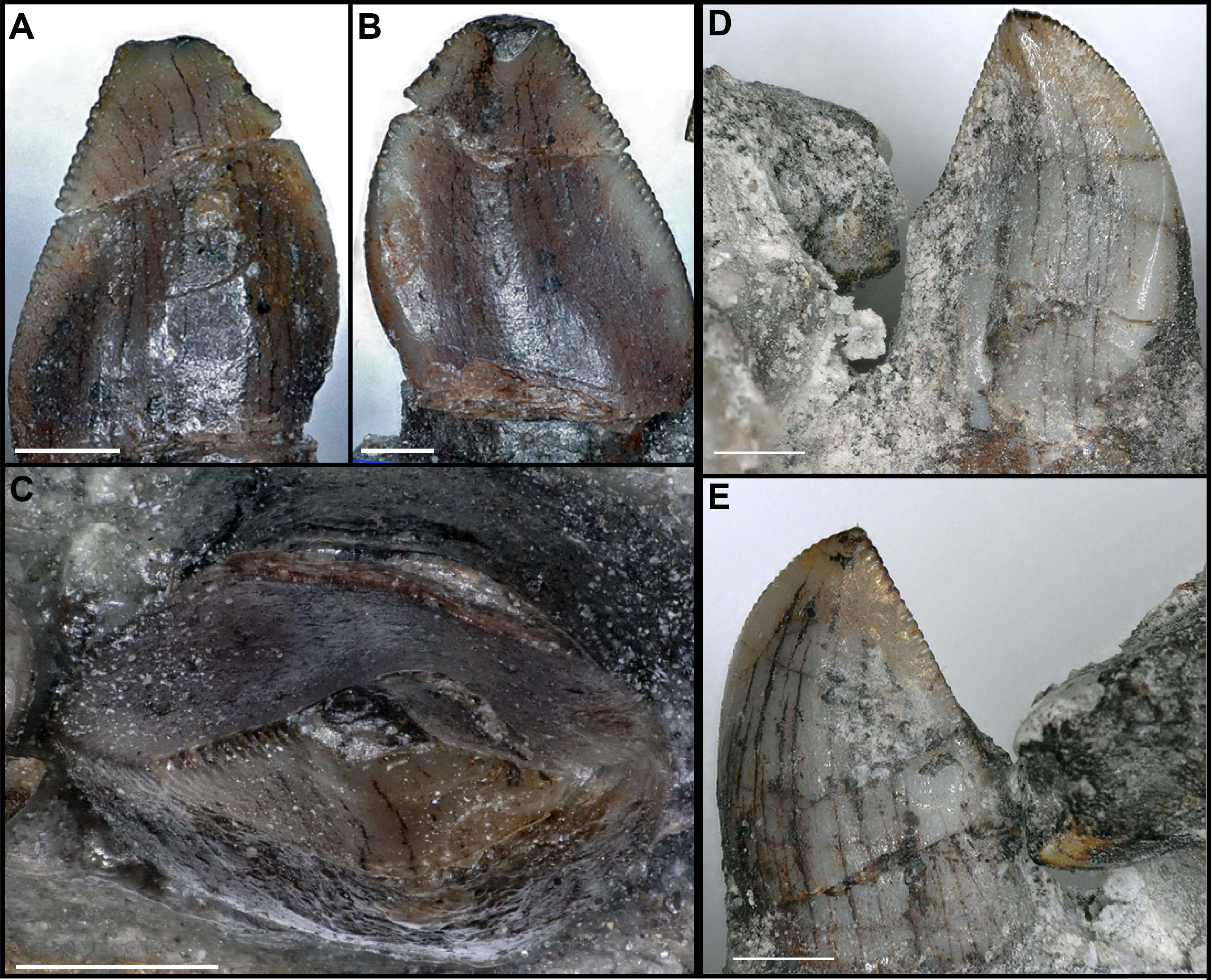

Supplement: Supplemental Information 2 — UMNH VP 14527 (A–C) and UMNH VP 15259 (D, E) in labial (A, D), lingual (B, E) , and occlusal (C) views. Scale bar represents 1 mm. [file peerj-05-4129-s002.png]

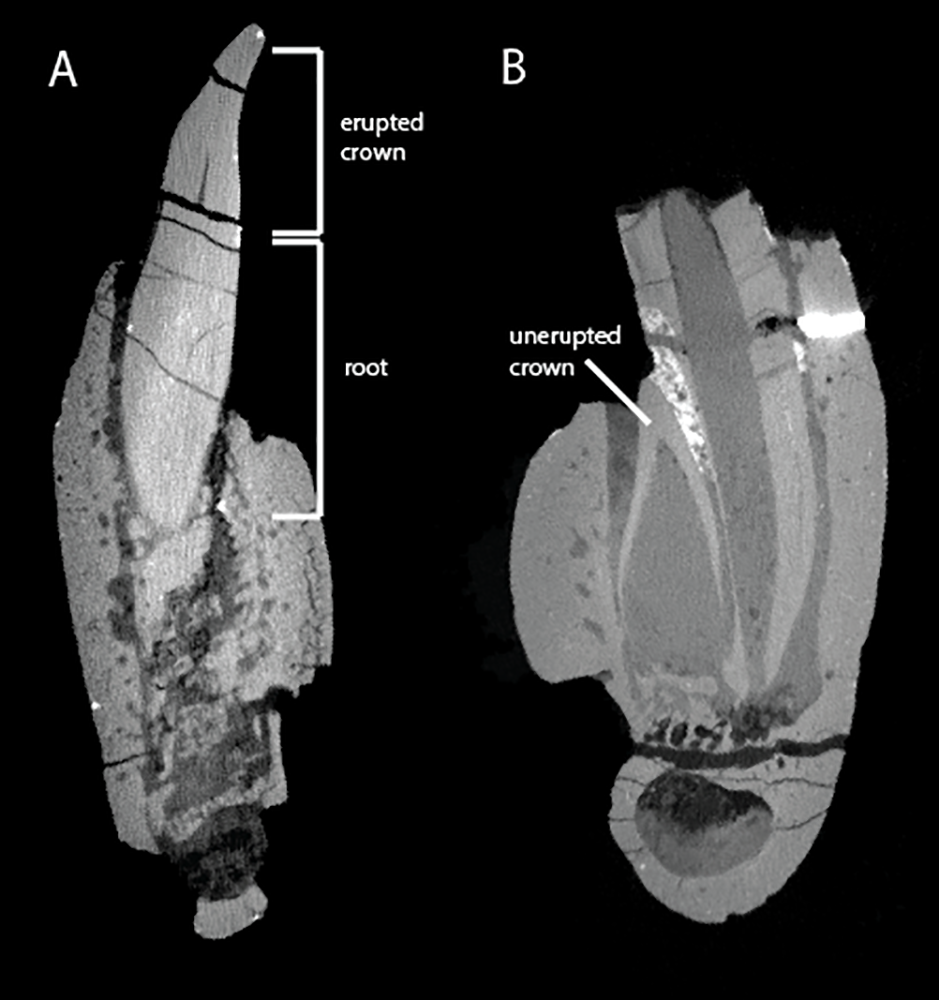

Supplement: Supplemental Information 3 — (A) UMNH VP 14527 in transverse (labiolingual) cross-section. (B) UMNH VP 14529 in transverse cross-section. Crowns are not completely formed until they have begun erupting from the alveolus. Root lengths that equal or exceed crown height are indicative of fully erupted functional teeth. [file peerj-05-4129-s003.png]
